# Abiraterone *In Vitro* Is Superior to Enzalutamide in Response to Ionizing Radiation

**DOI:** 10.3389/fonc.2021.700543

**Published:** 2021-07-21

**Authors:** Timothy C. Wright, Victoria L. Dunne, Ali H. D. Alshehri, Kelly M. Redmond, Aidan J. Cole, Kevin M. Prise

**Affiliations:** ^1^ Patrick G. Johnston Centre for Cancer Research, Queen’s University Belfast, Belfast, United Kingdom; ^2^ Department of Radiological Science, College of Applied Medical Sciences, Najran University, Najran, Saudi Arabia; ^3^ Northern Ireland Cancer Centre, Belfast Health & Social Care Trust, Belfast, United Kingdom

**Keywords:** prostate cancer, radiotherapy, androgen receptor, DNA damage, abiraterone, enzalutamide

## Abstract

Abiraterone acetate and Enzalutamide are novel anti-androgens that are key treatments to improve both progression-free survival and overall survival in patients with metastatic castration-resistant prostate cancer. In this study, we aimed to determine whether combinations of AR inhibitors with radiation are additive or synergistic, and investigated the underlying mechanisms governing this. This study also aimed to compare and investigate a biological rationale for the selection of Abiraterone versus Enzalutamide in combination with radiotherapy as currently selection is based on consideration of side effect profiles and clinical experience. We report that AR suppression with Enzalutamide produces a synergistic effect only in AR-sensitive prostate models. In contrast, Abiraterone displays synergistic effects in combination with radiation regardless of AR status, alluding to potential alternative mechanisms of action. The underlying mechanisms governing this AR-based synergy are based on the reduction of key AR linked DNA repair pathways such as NHEJ and HR, with changes in HR potentially the result of changes in cell cycle distribution, with these reductions ultimately resulting in increased cell death. These changes were also shown to be conserved in combination with radiation, with AR suppression 24 hours before radiation leading to the most significant differences. Comparison between Abiraterone and Enzalutamide highlighted Abiraterone from a mechanistic standpoint as being superior to Abiraterone for all endpoints measured. Therefore, this provides a potential rationale for the selection of Abiraterone over Enzalutamide.

## Introduction

Despite recent advances, prostate cancer continues to represent the most common form of cancer and the second most common cause of cancer-related death among men globally (1). Normal maintenance and development of the prostate is dependent on androgens and androgen receptor (AR) signaling, which also plays a key driving role in the development and progression of prostate cancer (2). However, although chemical castration is initially effective, progression to a castration-resistant setting occurs in a significant number of cases (3).

Metastatic castration-resistant prostate cancer (MCRPC) represents the lethal form of the disease with a number of interventions leading to improved overall survival. Two such interventions are Abiraterone acetate (Abi) and Enzalutamide (Enz), second-generation ADT agents that have been shown to lead to increased overall and progression-free survival (4, 5). Abiraterone acts as an indirect AR inhibitor through inhibition of Cytochrome p450- α-hydroxylase/17,20-lyase (CYP17A1), a key enzyme in the androgen biosynthetic pathway (6), while Enzalutamide acts as a direct AR inhibitor with multiple mechanisms, such as acting as an AR antagonist, preventing translocation of the AR and inhibiting the binding of the AR to DNA (7).

As with ADT, radiation continues to represent a key treatment of locally advanced and metastatic prostate cancer. However, radioresistance continues to represent a major hurdle in a clinical setting (8), making combinations of radiotherapy with additional therapeutics such as ADT an attractive option to help enhance outcomes. While combinations of ADT and radiotherapy have been shown to enhance clinical outcome (9–11), it is not known whether these effects are additive or synergistic. Recent studies have suggested the AR regulates a network of key DNA repair genes, providing a potential mechanism by which androgen deprivation may synergise with radiotherapy for prostate cancer (12, 13). Due to COVID-19, clinicians may opt to use abiraterone or enzalutamide in the up-front *de novo* metastatic setting as an alternative to the more immunosuppressive docetaxel chemotherapy. As such, increasing numbers of patients will be treated with radiotherapy and concomitant novel hormonal agents.

Treatment with ionizing radiation leads to the induction of DNA double-strand breaks (DSBs), which are repaired *via* two main mechanisms, Non-homologous end joining (NHEJ) and Homologous recombination (HR). NHEJ can occur at any stage of the cell cycle but is more error-prone. It involves the recruitment of the Ku70/80 heterodimer which acts as a scaffolding for the recruitment of other NHEJ repair factors such as DNA-dependent protein kinase catalytic subunit (DNA-PKcs) (14). HR requires a homologous template and so is restricted to the S and G2 phases of the cell cycle. It utilizes a core set of proteins, most notably Rad51 to catalyse key reactions with several other key factors (15). The AR has been shown to upregulate these key factors of DNA repair, although whether this is direct is still yet to be fully understood (12, 13). Suggesting that AR inhibition could play an important role in enhancing response to radiation.

Despite the clinical success of both Abiraterone acetate and Enzalutamide and both drugs achieving similar cancer control, there currently exists no biological rationale for the selection of one over the other, leaving the choice of therapy, a consideration of side effect profiles and clinical experience. Here we provide a direct comparison of the radiosensitizing potential of Abiraterone and Enzalutamide resultant of direct and indirect impacts on key DNA repair pathways such as NHEJ and HR and the significant benefit of Abiraterone over Enzalutamide across all metrics in an *in vitro* setting.

## Materials and Methods

### Cell Lines

Two human prostate cell lines were used: the hormone insensitive PC3 and the hormone-sensitive LNCaP. One osteoblastic cell model was used SJSA-1. All cell lines were obtained from ATCC (Manassas, Virginia, USA). PC3s, LNCaPs and SJSA-1s were grown in RPMI 1640 media [Thermo Fisher (Waltham, Massachusetts, USA)], supplemented with 10% Fetal bovine serum (FBS) (Thermo Fisher) and 50 µg/ml penicillin/streptomycin (Thermo Fisher).

### Antibodies

Antibodies were used according to manufacturer instructions. PARP [#9542, Santa Cruz (Dallas, Texas, USA)], PSA/KLK3 [D6B1, Cell Signalling (Danvers, Massachusetts, USA)], Rad51 (sc-398587, Santa Cruz), DNA-PK [ab70250, Abcam (Cambridge,UK)] and β-Actin (C4: sc-47778) primary antibodies were used in conjunction with HRP conjugated mouse and rabbit secondary antibodies (Life Technologies, USA).

### Irradiation

Cells were irradiated across various doses at 225kVp,13.3 mA in an X-Rad 225 Radiation cabinet (Precision X-RAY Inc, North Branford, CT, USA). A constant dose rate of 0.55 Gy/min was used.

### MTT Assay

Cells were treated in 96 well plates with a dose range of 10 nM to 100 μM Abiraterone, Enzalutamide or DMSO control for 72 hours, after which 20 μl of 3-(4,5-Dimethylthiazol-2-yl)-2,5-Diphenyltetrazolium Bromide (MTT) dye was added and left for a period of up to two hours. The solution was then removed and 100 μl DMSO added to allow the formazan product to dissolve. The absorbance was measured at 570 nm immediately in a FLUOstar Omega plate reader. LD25 values were determined from MTT curves and indicate the drug concentration at which cell viability was reduced by 25% of that of the DMSO control cells.

### Colony Formation Assay

Colony formation assays were carried out according to published methods (16). Cells were pre-treated with 10μM of Abiraterone, Enzalutamide or DMSO two hours before radiation and drug incubation continued until stained. Cells were irradiated over a dose range of 0-8 Gy. Plating efficiency (PE) and survival fraction (SF) were calculated with the following equations:

PE=(number of colonies formed/number of cells seeded)×100%

SF=number of colonies formed after irradiation/(number of cells seeded×PE/100)

Sensitising enhancement ratio (SER) was calculated as the radiation dose needed for radiation alone divided by the dose needed for DMSO, Abiraterone or Enzalutamide at a survival fraction of 10%. Radiosensitization was determined through normalizing to drug-treated controls.

### Western Blotting

Cells were pre-treated with 10μM of Abiraterone, Enzalutamide or DMSO one or 24 hours before radiation. Following radiation, cells were harvested and extracted according to published methods at predetermined time-points (17). 40 µg samples were loaded onto Invitrogen NuPAGE 8% Bis-Tris Midi gels and after electrophoresis transferred onto Invitrogen IBlot2 regular stacks and transferred using an IBlot. The membranes were then blocked with 5% non-fat dairy milk in PBS-Tween (PBS-T; 10 mM sodium phosphate, 0.15M NaCl, 0.05% Tween-20, pH 7.5) and incubated overnight at 4°C with the corresponding primary antibodies. After washing with PBS-T membranes were incubated in their secondary antibodies at room temperature for two hours. The membranes were then washed, developed by ECL reagent (7.5ml Tris HCl, 16.5µl coumaric acid, 37.5µl luminol, 2.5µl H2O2) and visualized, before being probed again if required.

### Immunofluorescence

Cells were pre-treated with 10μM of Abiraterone, Enzalutamide or DMSO 24 hours before irradiation. Following irradiation, cells were permeabilized (0.5% of Triton X-100 in PBS) and fixed at pre-determined time points before being blocked in blocking buffer (5% FBS in PBS) and stained with 53BP1 primary antibody (1:5000) [NB100-304, Novus Biologicals (Colorado, USA)] for one hour before being washed four times and stained with Alexa Fluor 568 goat anti-rabbit IgG secondary antibody (1:2000) [A21429, Invitrogen (Massachusetts, USA)] in the dark for one hour. Following staining, cells were washed four times and mounted onto microscope slides using Prolong Gold antifade reagent with DAPI [P36930, Invitrogen (Massachusetts, USA)].

### Cell Cycle Analysis

Cells were pre-treated with 10μM of Abiraterone, Enzalutamide or DMSO one or 24 hours before radiation. Following radiation, cells were harvested at predetermined time-points before being suspended in 100% ice-cold ethanol. Samples were then centrifuged, resuspended in 1% FBS in PBS and excess ethanol removed before resuspending pellets in 360μl of PI/RNaseA. Samples were incubated at 37°C for 30 minutes before being analyzed by flow cytometry on a BD Acuri C6 Plus Flow Cytometer (San Jose, CA, USA).

### Statistical Analysis

All experiments were performed in triplicate. Unpaired students t-test was used for comparisons between two groups. All statistics and graph plotting used GraphPad 8.0 (GraphPad, La Jolla, CA, USA).

## Results

### Impact of Abiraterone and Enzalutamide on Cell Growth

**Figure 1:**
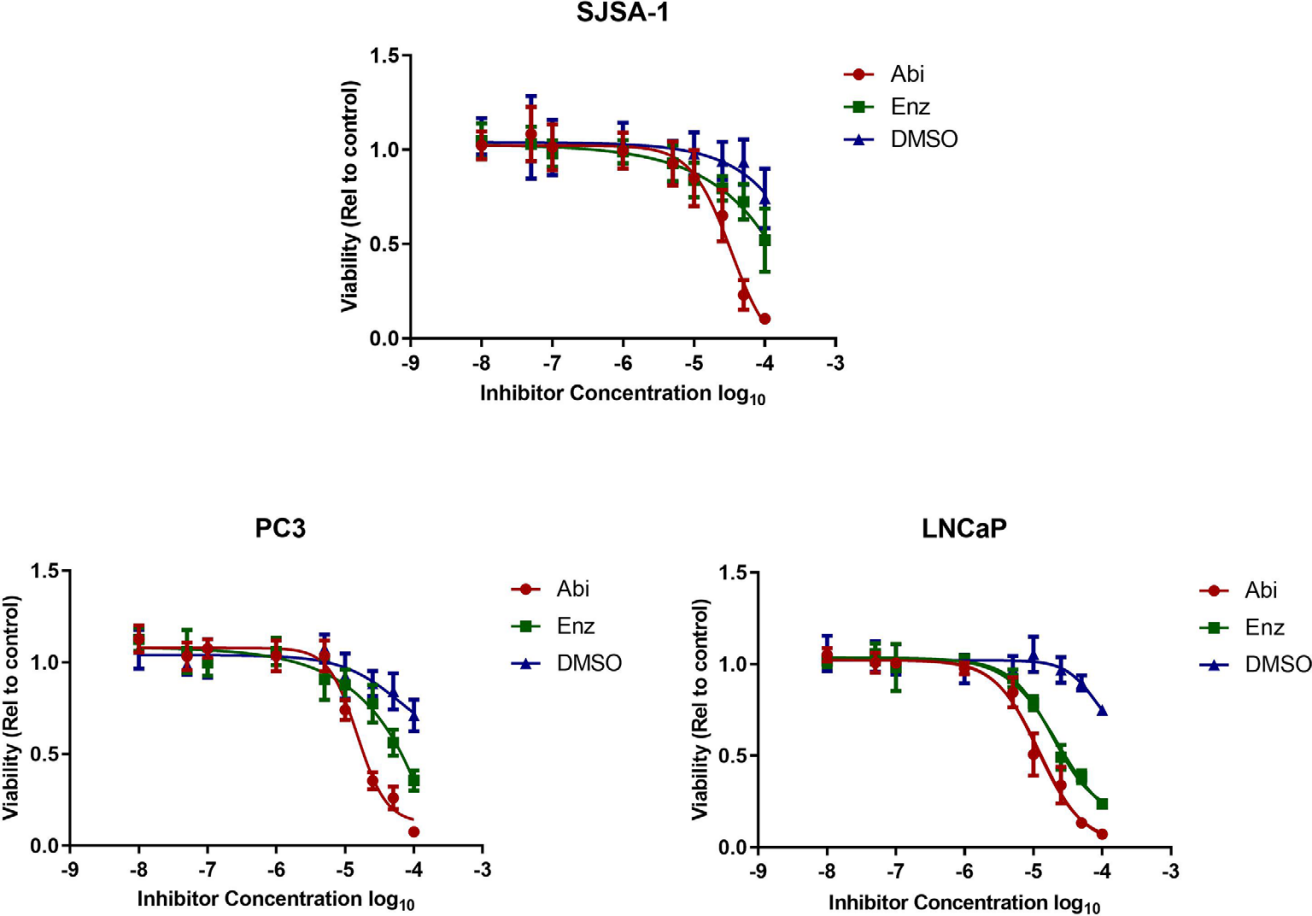
Effect of Abiraterone and Enzalutamide treatment on cell viability of androgen-sensitive (LNCaP), androgen-insensitive (PC3) prostate cancer models and osteoblastic bone model (SJSA-1). LNCaP, PC3, and SJSA-1 cells were treated with a dose range of 10 nM to 100 μM of Abiraterone, Enzalutamide or DMSO. Cell viability was evaluated 72 hours post-treatment by MTT assay. Each value is the mean of three independent experiments performed in triplicate and error bars represent SEM.

**Figure 2:**
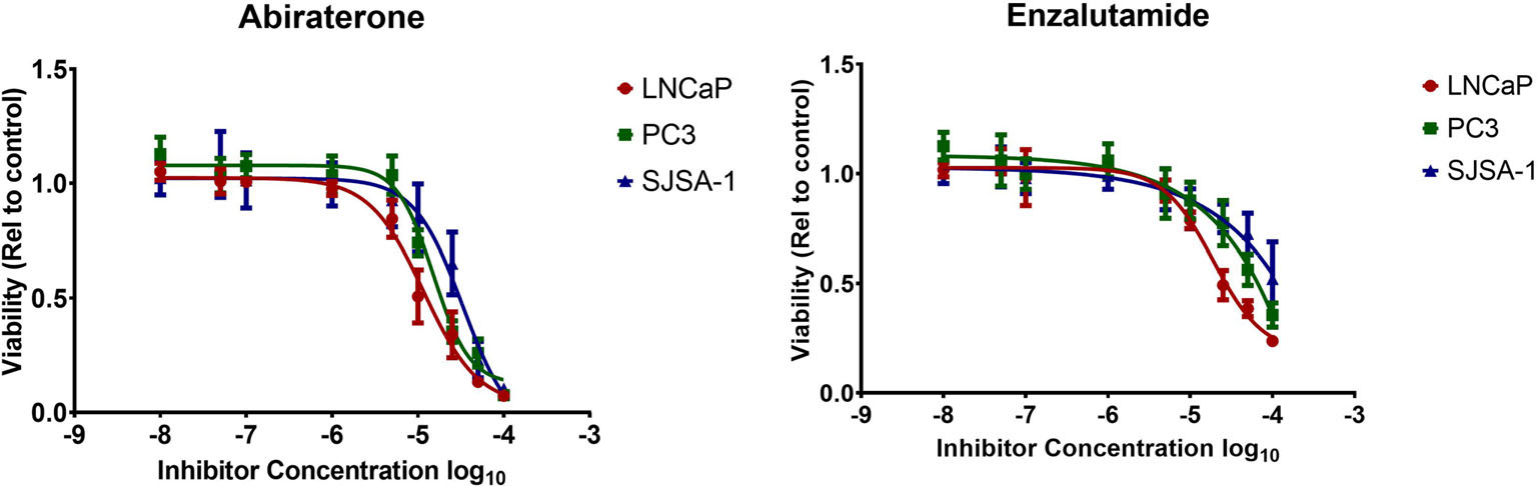
Comparison of Abiraterone and Enzalutamide treatment on cell viability of androgen-sensitive (LNCaP), androgen-resistant (PC3) prostate cancer models and osteoblastic bone model (SJSA-1). LNCaP, PC3, SJSA-1 cells were treated with a dose range of 10 nM to 100 μM of Abiraterone and Enzalutamide or DMSO. Cell viability was evaluated 72 hours post-treatment by MTT assay. Each value is the mean of three independent experiments performed in triplicate (errors represent SEM).

The cytostatic/cytotoxic effect of Abiraterone and Enzalutamide was studied using androgen-sensitive (LNCaP), androgen-insensitive (PC3) prostate cancer models and an osteoblastic bone model (SJSA-1). Both Abiraterone and Enzalutamide were shown to reduce the viability of all cell lines compared to DMSO controls (**Figure 1** and **Table 1**). Direct comparison of all models to determine the effect of AR status (**Figure 2**) showed both PC3s and SJSA-1s displayed similar responses to both Abiraterone (LD25 = 12.6 µM and 16.2 µM) and Enzalutamide (LD25 = 23.4 µM and 34.7 µM) treatment across the dose range, while LNCaPs displayed increased sensitivity to Abiraterone and Enzalutamide compared to both PC3s and SJSA-1s and also showed increased sensitivity to Abiraterone (LD25 = 5.8 µM) over Enzalutamide (LD25 = 12 µM). Investigations into fold sensitivity increase over DMSO (**Supplementary Table 1**) showed PC3s and SJSA-1s displayed similar fold sensitivity increases over DMSO for both Abiraterone (both 6.6) and Enzalutamide (3.5 and 3.1), while the androgen sensitive LNCaPs were more sensitive to Abiraterone (15.8) and Enzalutamide (7.6) as expected.

**Table 1:** LD25 values of MTT values across cell lines ± SEM. LD25 values were determined from MTT curves and indicate the drug concentration at which cell viability was reduced by 25%.

LD25 (µM)	Treatment	LNCaP	SJSA-1	PC3
	DMSO	91.2 ± 0.0052	107.15 ± 0.059	83.18 ± 0.072
	Enz	12.02 ± 0.051	34.67 ± 0.062	23.44 ± 0.061
	Abi	5.75 ± 0.053	16.22 ± 0.049	12.59 ± 0.043

### Is the Addition of Radiotherapy to Abiraterone or Enzalutamide Synergistic or Additive?

**Figure 3:**
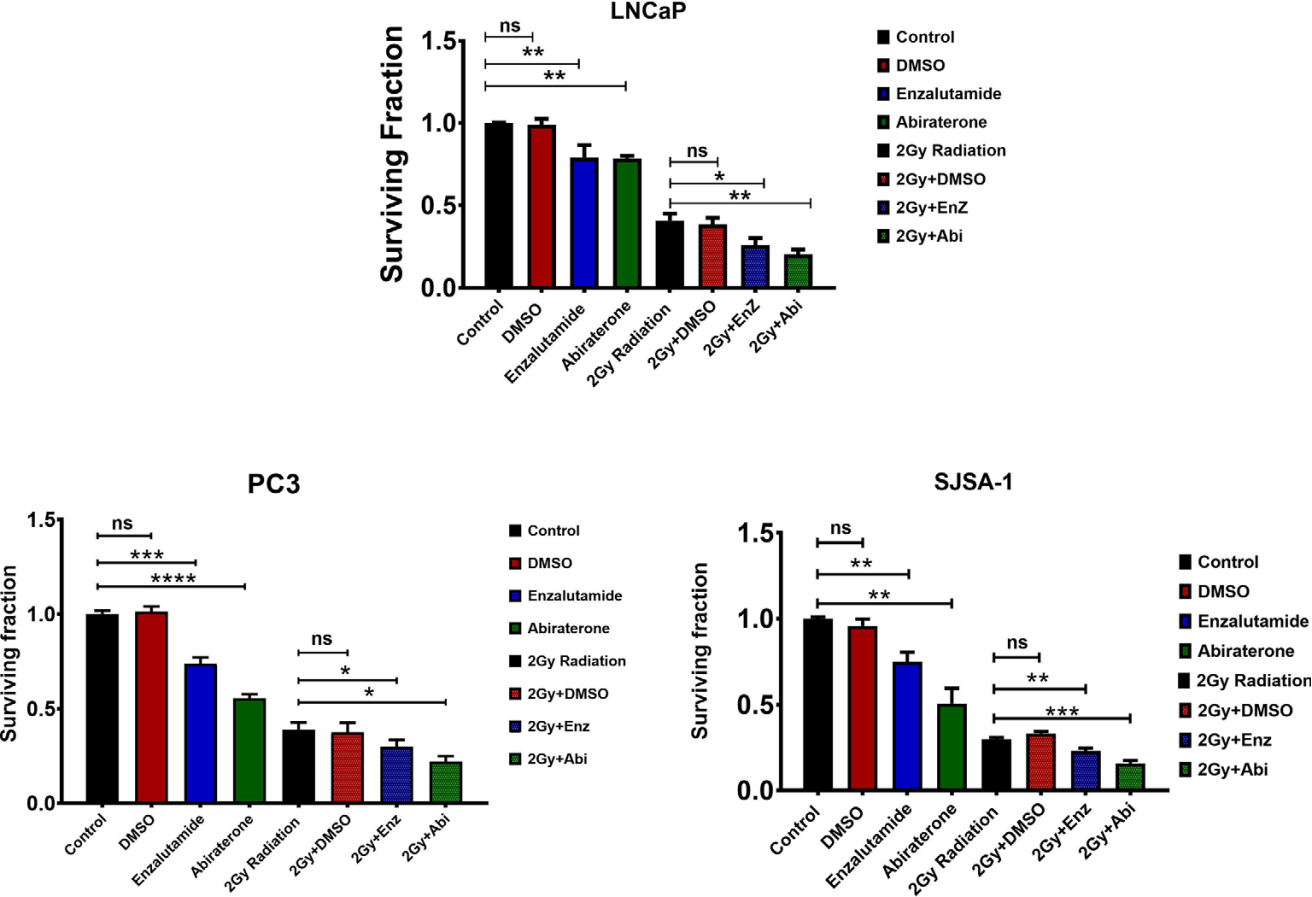
Comparison of the combined effect of Abiraterone and Enzalutamide and DMSO as single agents or combined with 2 Gy radiation on survival fraction in androgen-sensitive LNCaPs and androgen insensitive PC3s prostate cancer models and osteoblastic bone model SJSA-1. PC3s and SJSA-1s were treated with 10 μM Abiraterone, Enzalutamide or DMSO 24 hours before radiation, while LNCaPs were treated with 200 nM due to their sensitivity. Cells were then left an appropriate amount of time to form sufficient colonies and any colonies of 50 cells or more counted. Each value is the mean of three independent experiments performed in triplicate (+/- SEM) and normalized to control. Unpaired students t-test was used for comparisons between two groups. *p ≤ 0.05, **p ≤ 0.01, ***p ≤ 0.001, ****p ≤ 0.0001, ns= non significant.

With both Enzalutamide and Abiraterone being shown to improve survival in an MCRPC setting, there, therefore, exists a biological rationale that their combination with radiotherapy could exceed that of their use as a monotherapy, which was investigated through use of clonogenic survival assays. For clonogenic survival (**Figure 3**), while DMSO showed little to no additive impact on survival fraction, both Enzalutamide (PC3: ***P ≤ 0.001, ≤ 0.0001, SJSA-1: **P ≤ 0.01 and LNCaP: **P ≤ 0.01) and Abiraterone (PC3: ****P ≤ 0.0001, SJSA-1: **P ≤ 0.01 and LNCaP: **P ≤ 0.01) as single agents were shown to significantly affect the survival fraction of all models, irrespective of AR status. Comparison 2 Gy radiation to 2 Gy radiation in combination with Enzalutamide (PC3: *P ≤ 0.05, SJSA-1: **P ≤ 0.01 and LNCaP: *P ≤ 0.05) or Abiraterone (PC3: *P ≤ 0.05, SJSA-1: ***P ≤ 0.001 and LNCaP: **P ≤ 0.01) showed significant additive effects across all models regardless of AR status.

**Figure 4:**
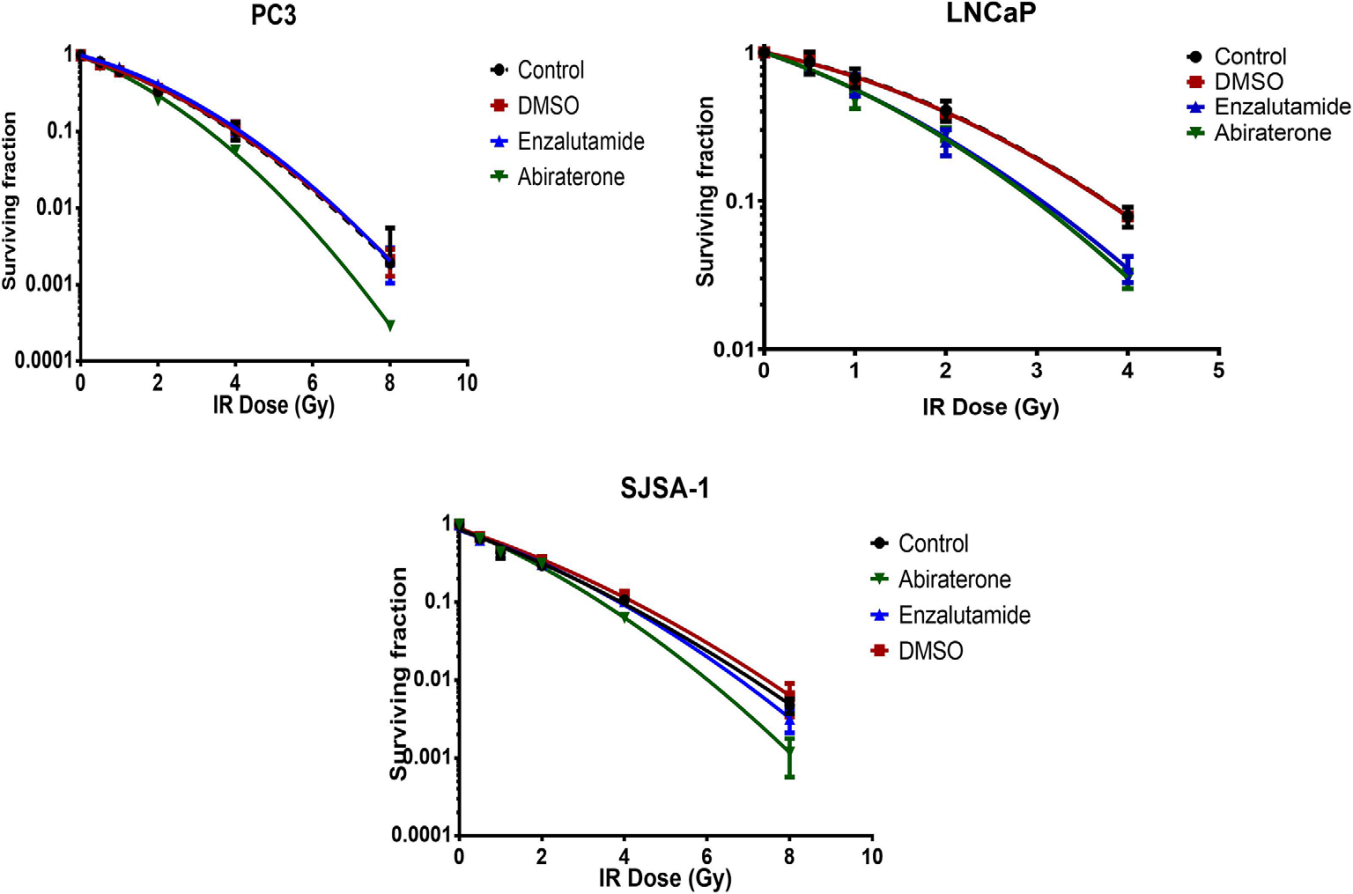
Radiosensitization effects of Abiraterone, Enzalutamide and DMSO on radiation in androgen-insensitive PC3 prostate cancer model, androgen-sensitive LNCaP model and osteoblastic bone model SJSA-1 by colony formation assay. LNCaPs were treated with 200 nM, while PC3s and SJSA-1s were treated with 10 μM Abiraterone, Enzalutamide or DMSO 24 hours before X-Ray across a dose range of 0-8 Gy. Cells were then left to form appropriately sized colonies and survival fraction calculated using SF = (colonies counted) / (cells seeded x (PE/100) colonies counted). Error bars are standard error of the mean (+/- SEM) and for some points, the error bars are shorter than the height of the symbol (n=3).

To determine synergistic effects (i.e. whether the combination of Abiraterone or Enzalutamide with radiation is greater than combined individual toxicity), clonogenic survival assays were normalized to account for the additive drug-mediated cytotoxicity that had been observed previously, therefore allowing examination of only radiation-induced effects on proliferation (**Figure 4** and **Table 2**). LNCaPs showed increased radiosensitivity when pre-treated 24 hours before radiation with both Abiraterone (SER=1.23) and Enzalutamide (SER=1.23), while no radiosensitizing effects were observed with Enzalutamide in both PC3s (SER=0.96) and SJSA-1s (SER=1.01). Abiraterone displayed synergy with radiation in AR resistant PC3s (SER=1.19) and SJSA-1s (SER=1.17).

**Table 2:** SER values of inhibitors *vs* control at 10% with +/- SEM.

Cell line	DMSO	Enz	Abi
PC3	0.98 ± 0.042	0.96 ± 0.049	1.19 ± 0.045
LNCaP	1 ± 0.092	1.23 ± 0.069	1.23 ± 0.075
SJSA-1	0.94 ± 0.019	1.00 ± 0.022	1.16 ± 0.022

Sensitising enhancement ratio (SER) was calculated as the radiation dose needed for radiation alone divided by the dose needed for DMSO, Abiraterone or Enzalutamide at a survival fraction of 10%.

### Impact of Abiraterone and Enzalutamide With or Without Radiotherapy on DNA Damage and Repair

**Figure 5:**
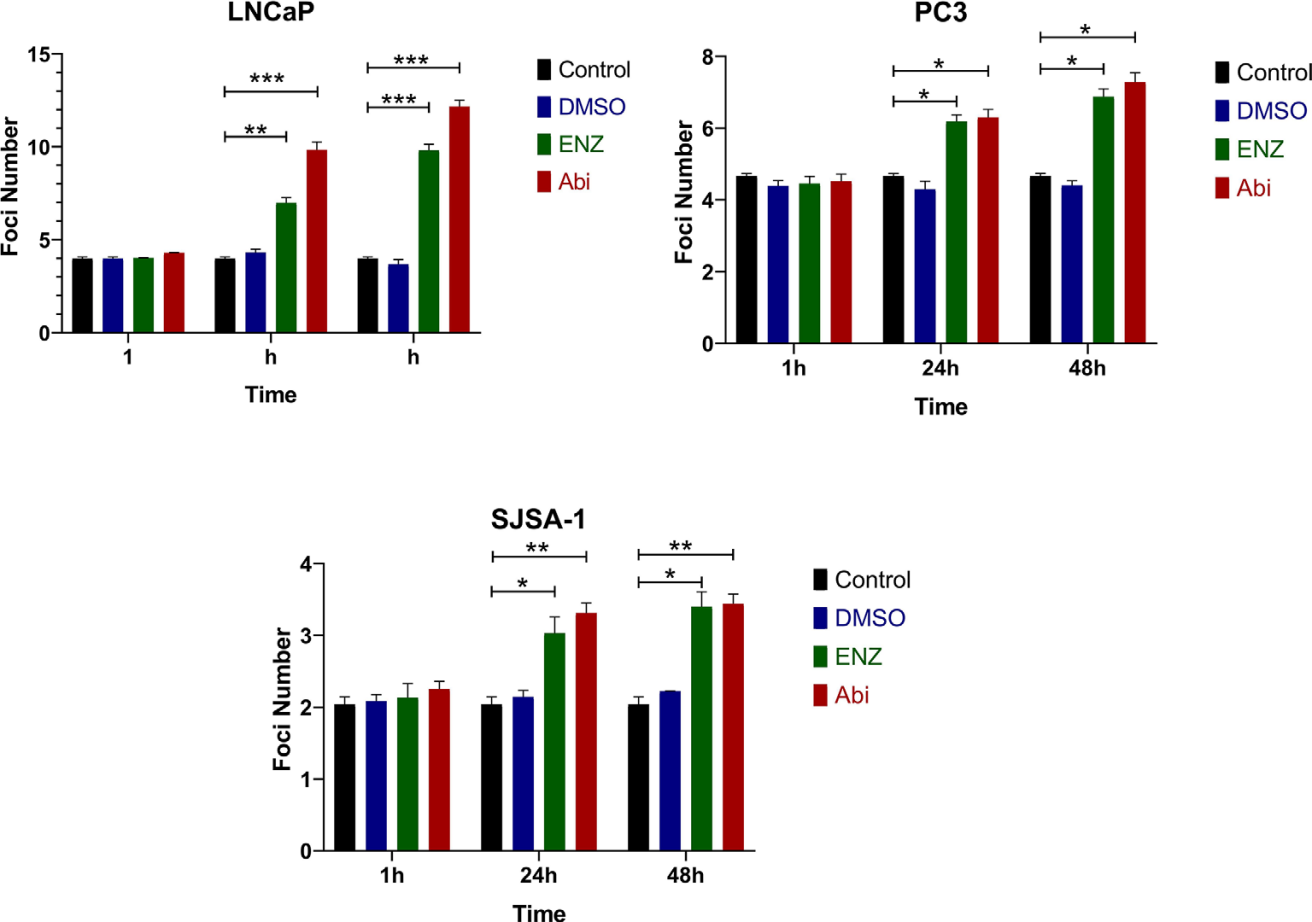
Immunofluorescence of 53BP1 foci treated with Abiraterone and Enzalutamide on AR-insensitive PC3s and AR-sensitive LNCaP prostate models and osteoblastic bone model SJSA-1. All models were treated with 10 μM Abiraterone, Enzalutamide or DMSO and harvested 1-, 24 and 48-hours post-treatment before being fixed and stained with 53BP1 (n=3). Unpaired students t-test was used for comparisons between two groups *p ≤ 0.05, **p ≤ 0.01, ***p ≤ 0.001, and error bars represent SEM.

The impact of Enzalutamide and Abiraterone on DNA damage was also assessed through quantifying changes in DSB levels by 53BP1 foci *via* immunofluorescence with and without 2Gy radiation (**Figure 5**). Treatment with either Abiraterone or Enzalutamide led to significant increases in DNA damage regardless of AR status 24 hours ((PC3 (*p ≤ 0.05 and *p ≤ 0.05), SJSA-1 (*p ≤ 0.05 and **p ≤ 0.01) and LNCaP (**p ≤ 0.01 and ***p ≤ 0.001)) and 48 hours post treatment ((PC3 (*p ≤ 0.05 and *p ≤ 0.05), SJSA-1 (*p ≤ 0.05 and **p ≤ 0.01) and LNCaP (***p ≤ 0.001 and ***p ≤ 0.001)).

**Figure 6:**
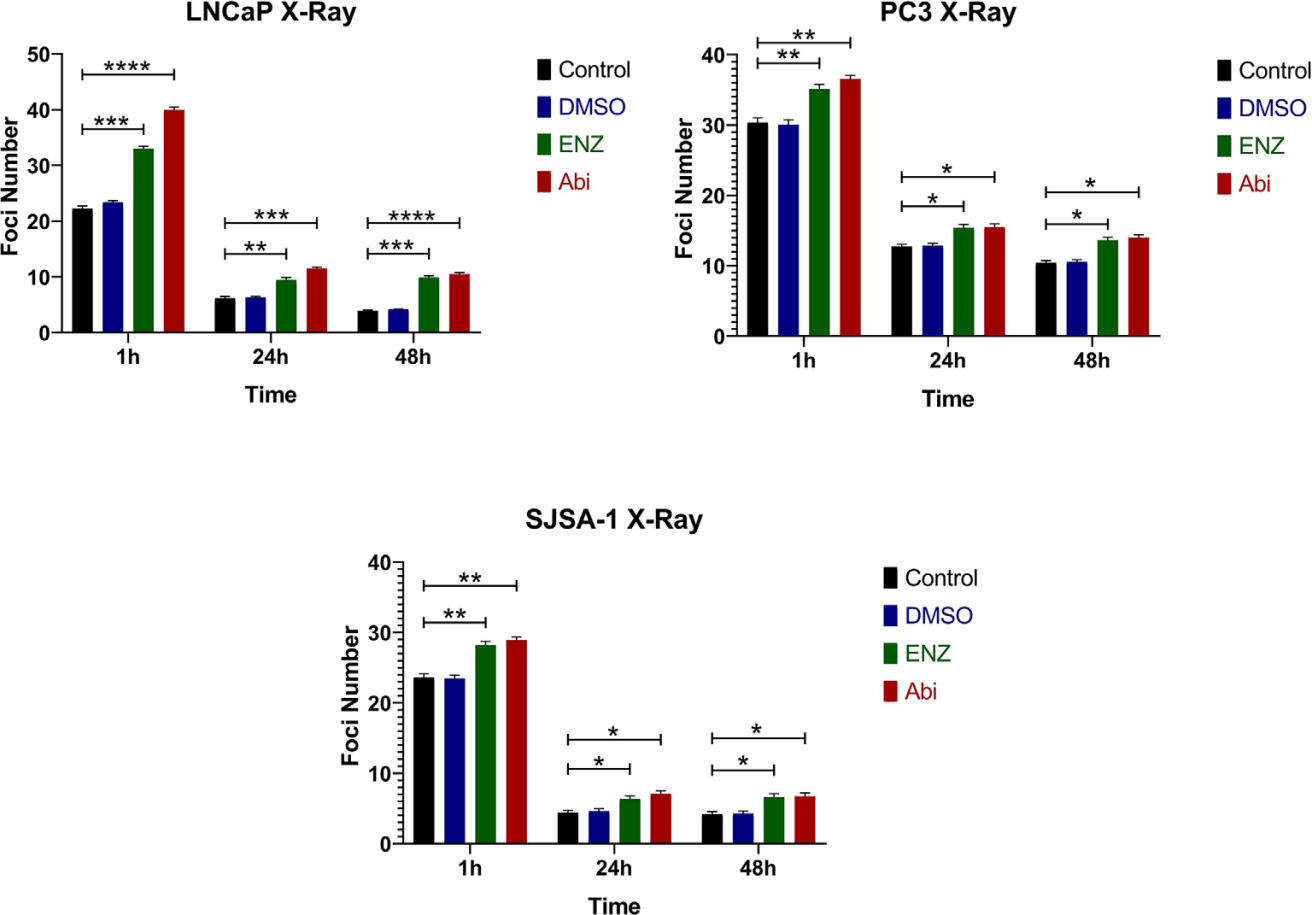
Immunofluorescence of 53BP1 foci treated with Abiraterone and Enzalutamide in combination with 2 Gy X-ray in AR-insensitive PC3s and AR-sensitive LNCaP prostate models and osteoblastic bone model SJSA-1. All models were treated with 10 μM Abiraterone, Enzalutamide or DMSO 24 hours before being administered 2 Gy radiation. Samples were then harvested 1, 24- and 48-hours post-radiation before being fixed and stained with 53BP1 (n=3). Unpaired students t-test was used for comparisons between two groups *p ≤ 0.05, **p ≤ 0.01, ***p ≤ 0.001 ****p ≤ 0.0001, and error bars represent SEM.

The impact of Enzalutamide and Abiraterone mediated DNA damage with radiation damage was also assessed, with cells irradiated with 2 Gy X-rays 24-hour post treatment with Abiraterone or Enzalutamide (**Figure 6**). As expected, irradiation alone led to large increases in 53BP1 foci, one hour post radiation when DNA damage levels were at their highest, which decreased in a time dependent manner. The addition of Abiraterone and Enzalutamide was shown to significantly enhance DNA damage one hour ((PC3 (**p ≤ 0.01 and **p ≤ 0.01), SJSA-1 (**p ≤ 0.01 and **p ≤ 0.01) and LNCaP (***p ≤ 0.001 and ****p ≤ 0.0001)), 24 hours ((PC3 (*p ≤ 0.05 and *p ≤ 0.05), SJSA-1 (*p ≤ 0.05 and *p ≤ 0.05) and LNCaP (**p ≤ 0.01 and ***p ≤ 0.001)) and 48 hours ((PC3 (*p ≤ 0.05 and *p ≤ 0.05), SJSA-1 (*p ≤ 0.05 and *p ≤ 0.05) and LNCaP (***p ≤ 0.001 and ****p ≤ 0.0001)) post irradiation.

### Impact of Abiraterone and Enzalutamide With or Without Radiotherapy on HR Repair

**Figure 7:**
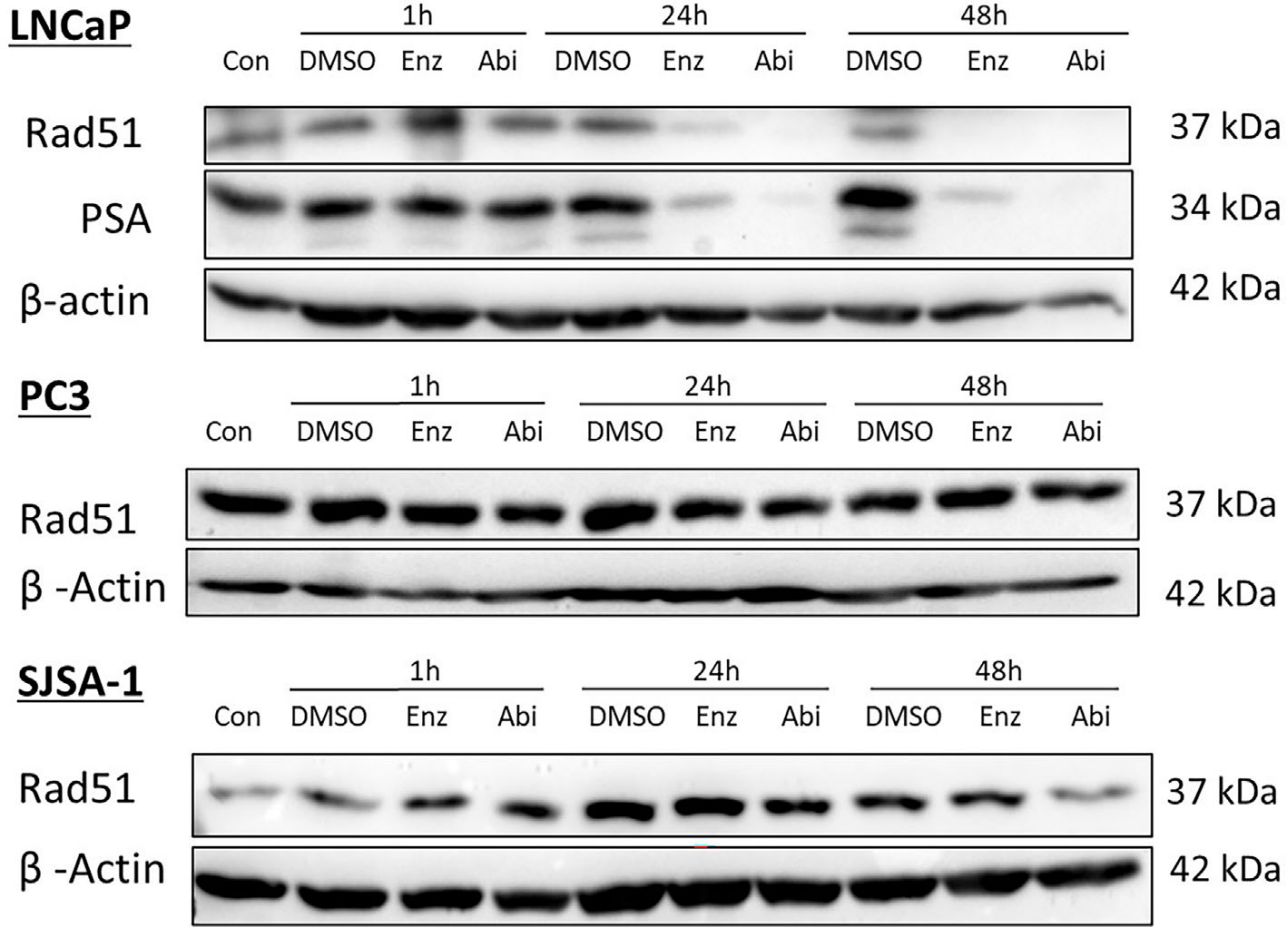
Impact of Abiraterone and Enzalutamide on RAD51 and PSA protein expression in AR-sensitive LNCaP prostate model, AR-insensitive PC3 prostate model and osteoblastic bone model SJSA-1. All models were treated with 10 μM Abiraterone, Enzalutamide or DMSO. Samples were then harvested 1, 24- and 48-hours post-treatment and expression levels measured *via* Western blot. β-Actin was used as a loading control. (n=3).

As previously described, the androgen receptor has been linked to the upregulation of key DNA repair genes. Therefore, the impact of AR suppression on HR was investigated through observations of RAD51 expression, a key component in mediating HR repair of DSBs. AR suppression with Abiraterone and Enzalutamide as single agents, as verified by showing a reduction in downstream PSA expression, directly correlated with a total visible reduction of RAD51 protein expression in LNCaPs (**Figure 7**). Supporting this is an AR-mediated effect, both PC3s and SJSA-1s showed no noticeable changes in RAD51 expression regardless of timepoint. PSA expression could not be measured in PC3, or SJSA-1 cells as they do no signal through their AR, resulting in no transcription of prostate-related proteins such as PSA (18). Comparisons between Abiraterone and Enzalutamide showed that while Abiraterone achieved a total reduction of RAD51 at an earlier timepoint than Enzalutamide, both achieved total reduction by 48 hours post-treatment.

**Figure 8:**
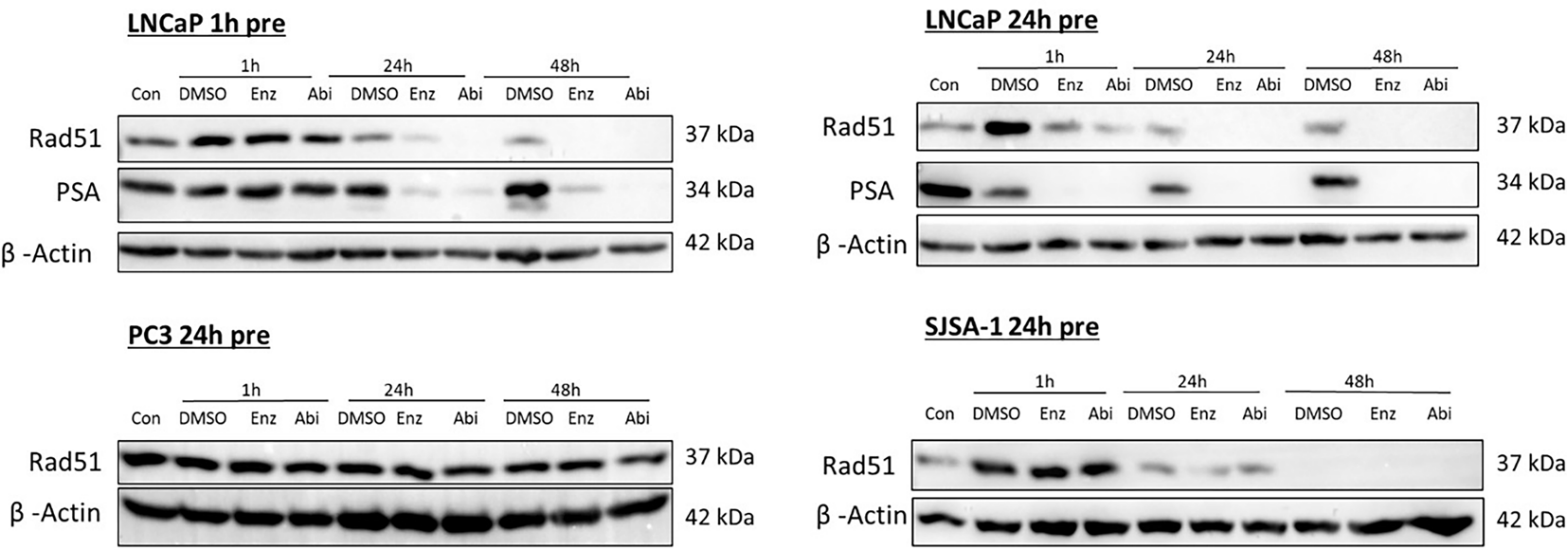
Impact of Abiraterone and Enzalutamide in combination with 2 Gy X-ray on RAD51 and PSA expression in AR-insensitive PC3s and AR-sensitive LNCaP prostate models and osteoblastic bone model SJSA-1. All models were treated with 10 μM Abiraterone, Enzalutamide or DMSO 1 or 24 hours before radiation. Samples were then harvested 1, 24 and 48 hours post-radiation and expression levels measured *via* Western blot. β-Actin was used as a loading control. (n=3).

Co-treatment of AR inhibitors with radiation was also investigated, to determine if these effects were conserved with radiation (**Figure 8**), with both 1- and 24-hour pre-treatment with Abiraterone or Enzalutamide before irradiation investigated to evaluate whether any effects were time-dependent. As observed when used as a monotherapy, pre-treatment with Abiraterone or Enzalutamide in an AR-sensitive setting before irradiation with 2 Gy led to non-detectable RAD51 protein levels. Pre-treatment with Enzalutamide or Abiraterone 24 hours before radiation treatment was shown to cause large reductions in RAD51 levels even one-hour post-radiation, where DNA damage is at its maximum and levels of RAD51 at their highest. Abiraterone showed increased depletion of RAD51 levels one hour post-radiation with 24 hour pre-treatment compared to Enzalutamide.

### Impact of Abiraterone and Enzalutamide With or Without RT on NHEJ Repair

**Figure 9:**
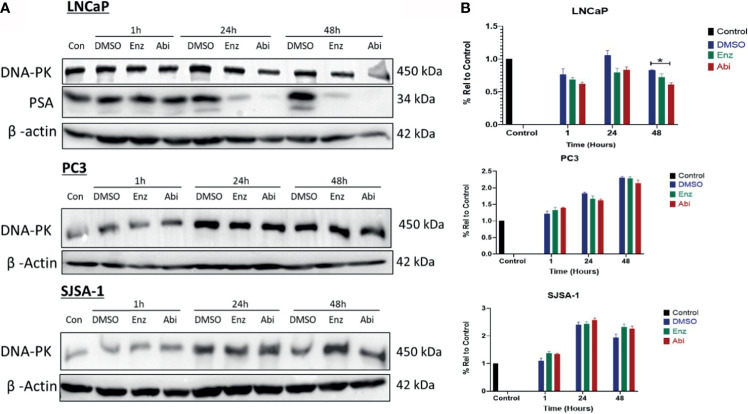
Impact of Abiraterone and Enzalutamide on DNA-PK and PSA protein expression in AR-sensitive LNCaP and AR-insensitive PC3 prostate models and osteoblastic bone model SJSA-1. All models were treated with 10 μM Abiraterone, Enzalutamide or DMSO. Samples were then harvested 1, 24 and 48 hours post-treatment and expression levels measured *via* Western blot **(A)** and densitometric analysis **(B)**. β -Actin was used as a loading control. (n=3). Unpaired students t-test was used for comparisons between two groups *p < 0.05 and error bars represent SEM.

**Figure 10:**
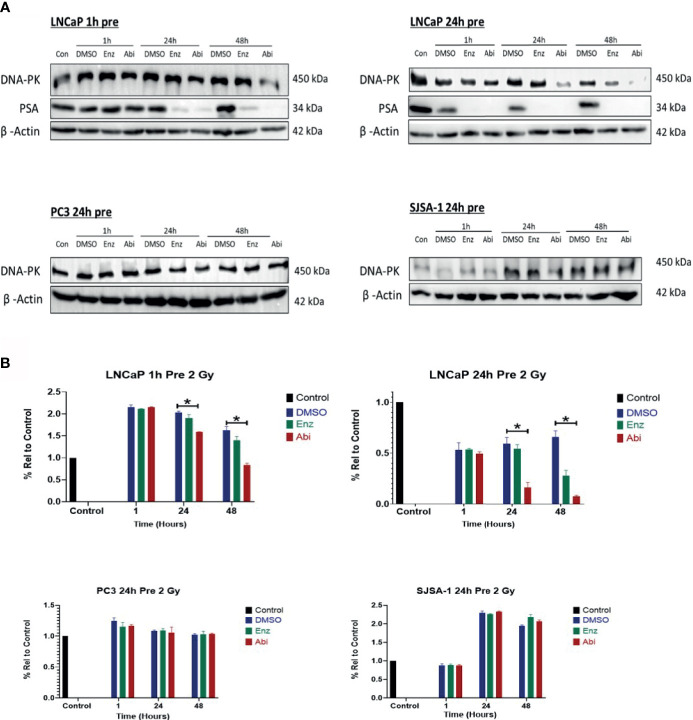
Impact of Abiraterone and Enzalutamide in combination with 2 Gy X-ray on DNA-PK and PSA expression in AR-insensitive PC3s, AR-sensitive LNCaP prostate models and osteoblastic bone model SJSA-1. All models were treated with 10 μM Abiraterone, Enzalutamide or DMSO for 1 or 24 hours before being administered 2 Gy radiation. Samples were then harvested 1, 24 and 48 hours post-radiation and expression levels measured *via* Western blot **(A)** and densitometric analysis **(B)**. β -Actin was used as a loading control. (n=3). Unpaired students t-test was used for comparisons between two groups *p < 0.05 and error bars represent SEM.

DNA-PK expression was also investigated to determine if the observed impacts of Abiraterone and Enzalutamide on HR extended to other forms of DSB repair such as NHEJ (**Figure 9**). DNA-PK levels were shown to reduce in a time-dependent manner correlating with PSA levels following treatment with both Enzalutamide and Abiraterone in LNCaPs, however, only 48 hour treatment with Abiraterone was shown to be significant upon statistical testing (*p ≤ 0.05). No significant changes in DNA-PK levels were observed in PC3s or SJSA-1s. Comparison of Abiraterone against Enzalutamide showed only Abiraterone caused significant reductions in DNA-PK levels (*p ≤ 0.05). Combinations of Abiraterone or Enzalutamide with 2 Gy X-ray radiation (**Figure 10**) showed enhanced reductions of DNA-PK levels than with inhibitors alone. Pre-treatment with Abiraterone for 1 to 24 hours before radiation treatment was shown to induce significant reductions in DNA-PK levels both 24 and 48 hours post-radiation (*p ≤ 0.05). Pre-treatment 24 hours prior to radiation showed larger observable reductions in DNA-PK levels then one hour pre-treatment.

### Impact of Abiraterone and Enzalutamide With or Without RT on Cell Cycle Distribution and Cell Death

**Figure 11:**
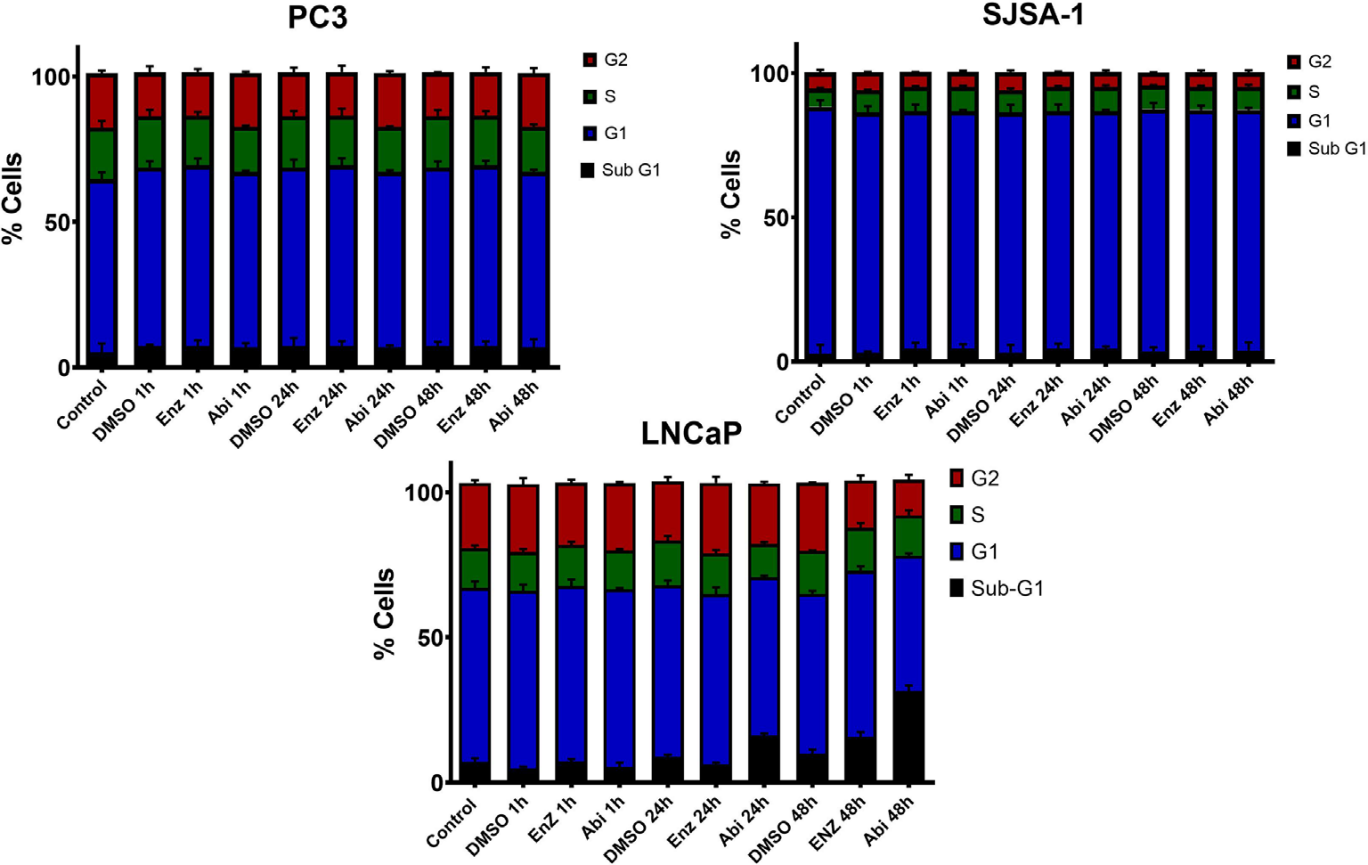
Cell-cycle analysis of AR-sensitive LNCaP prostate model, AR- insensitive PC3 prostate model and osteoblastic bone model SJSA-1. Cells were treated with 10 μM Abiraterone, Enzalutamide or DMSO, fixed in ice cold ethanol 1h, 24h and 48h post-treatment and stained with PI/RNaseA for 30 minutes before the cell-cycle profile was determined by flow cytometry. Error bars are standard error of the mean (SEM) (n=3).

The previous results have shown that AR suppression through Abiraterone and Enzalutamide has a significant impact on multiple DNA repair pathways involved in DSB repair. However, the choice of repair pathway is also dependent on which phase of the cell cycle the cell arrests in. Cell cycle distribution was therefore investigated to determine whether observed changes were due to the direct impact of these inhibitors on DNA repair genes, or indirectly through means of cell cycle distribution changes (**Figure 11**). Treatment with Enzalutamide and Abiraterone led to observed increases in sub-G1 levels in LNCaP cells, indicative of increased levels of apoptosis. With this effect shown to be more prominent with Abiraterone over Enzalutamide. Also evident were decreases in S and G2. These effects were not observed with PC3s and SJSA-1s.

**Figure 12:**
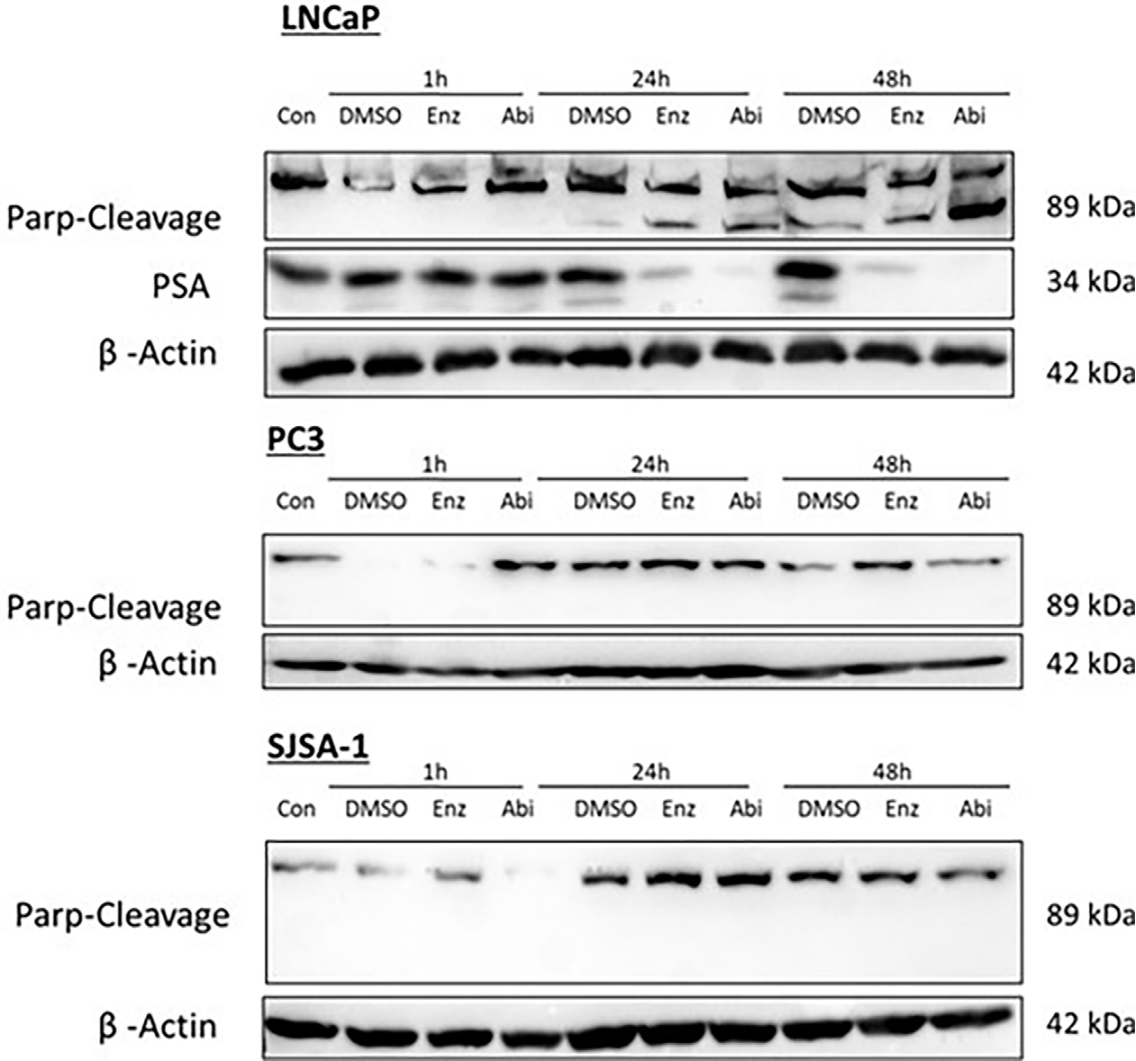
Impact of Abiraterone and Enzalutamide on PARP cleavage in AR-insensitive PC3s, AR-sensitive LNCaP prostate models and osteoblastic bone model SJSA-1. Models were treated with 10 μM Abiraterone, Enzalutamide or DMSO. Samples were then harvested 1, 24 and 48 hours post-treatment and expression levels measured *via* Western blot. β-Actin was used as a loading control. (n=3).

Potential increases in apoptosis as indicated by the increases in the sub-G1 population of cells were investigated through looking at the expression of PARP cleavage (**Figure 12**), with PARP cleavage by activated caspases being a defined hallmark of apoptosis. There was a correlation between increases in sub-G1 levels and PARP cleavage in LNCaPs, with both Abiraterone and Enzalutamide showing increased levels of PARP cleavage in a time-dependent manner. Treatment with Abiraterone led to higher levels of PARP cleavage compared to treatment with Enzalutamide. Treatment with Abiraterone or Enzalutamide in PC3s and SJSA-1s showed little to no observable impact on PARP-cleavage levels.

**Figure 13:**
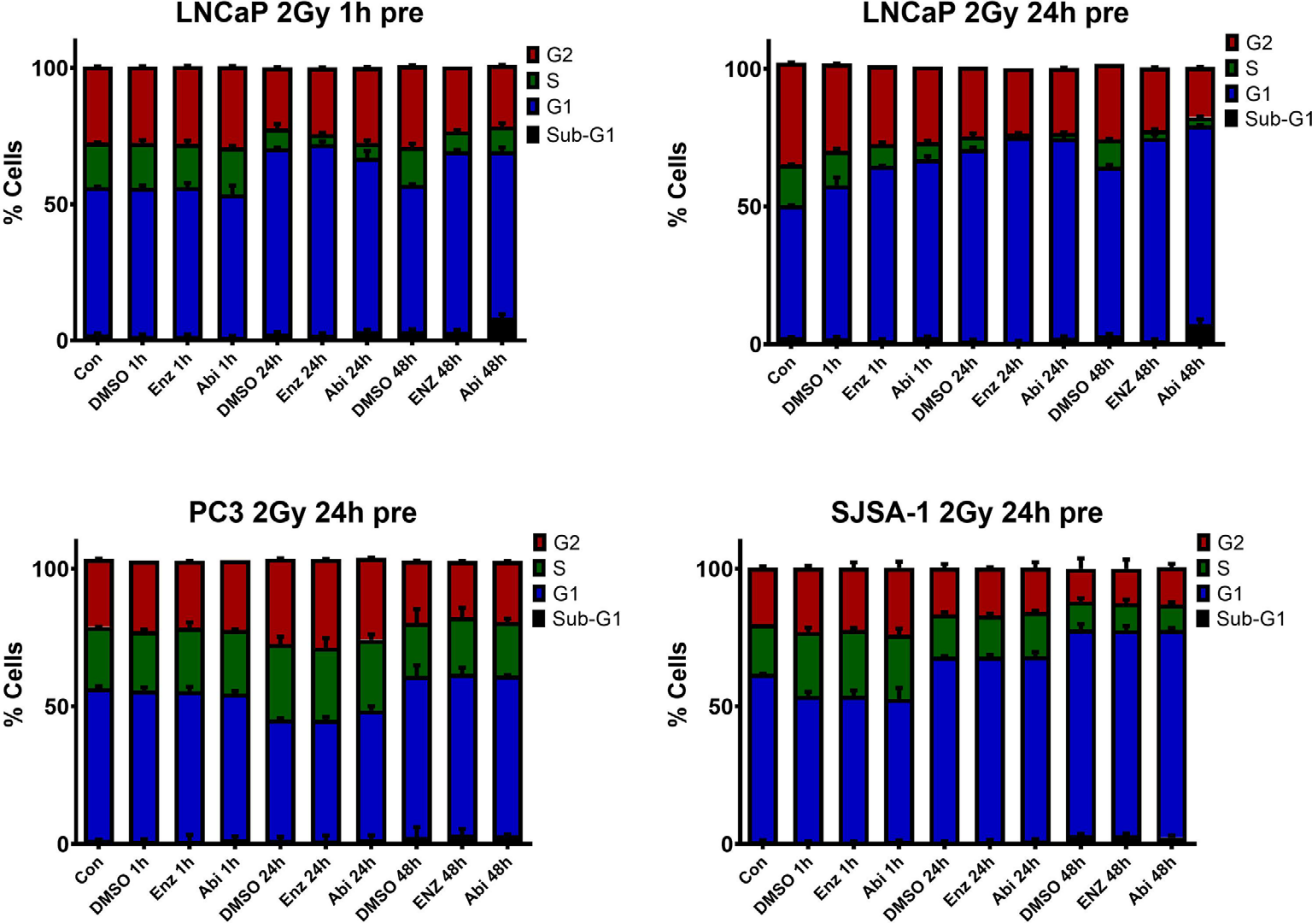
Cell-cycle analysis of AR-sensitive LNCaP prostate model, AR- insensitive PC3 prostate model and osteoblastic bone model SJSA-1. Cells were treated with 10 μM Abiraterone, Enzalutamide or DMSO 1 or 24 hours before radiation with 2 Gy. Post radiation cells were fixed in ice cold ethanol 1h, 24h and stained with PI/RNaseA for 30 minutes before the cell-cycle profile was determined by flow cytometry. Error bars are standard error of the mean (SEM) (n=3).

Combinations of Enzalutamide or Abiraterone with 2 Gy radiation (**Figure 13**) showed both effects to be conserved, with increased sub-G1 levels and decreased S and G2 levels shown in LNCaPs and no observable changes in PC3s and SJSA-1s. Pre-treatment 24h before radiation resulted in reductions in the proportions of cells in S and G2 phases one-hour post-radiation. This highlights the importance of ensuring AR-deprivation is achieved before radiation treatment over treating concurrently with radiation.

**Figure 14:**
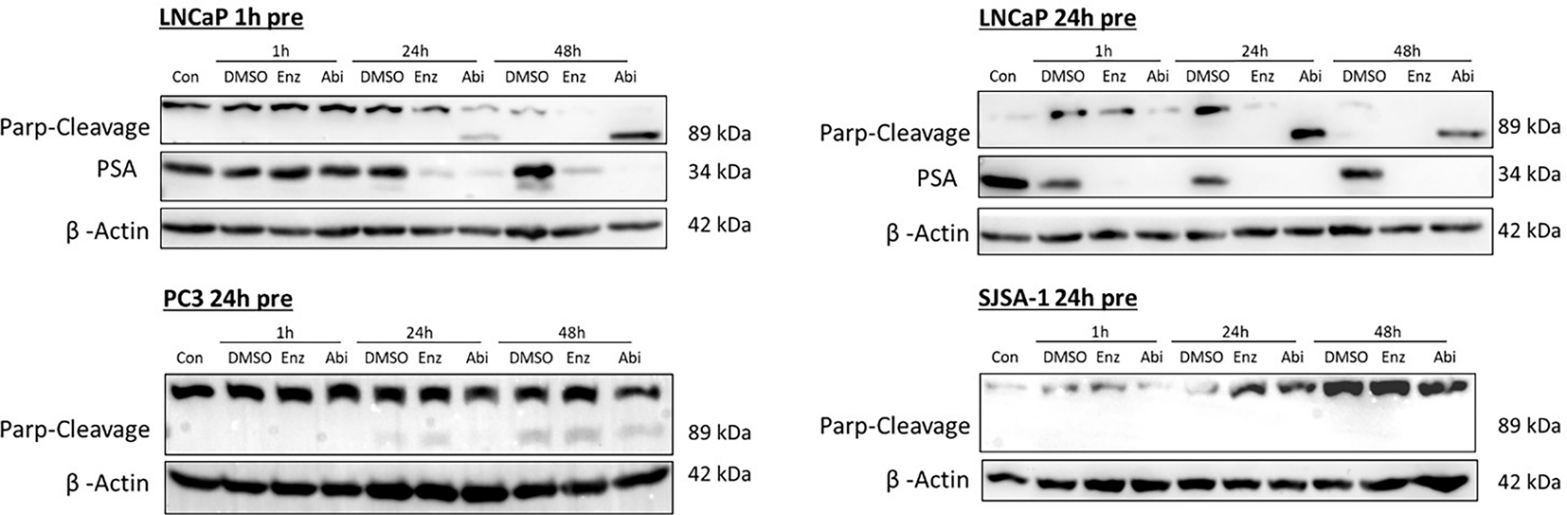
Impact of Abiraterone and Enzalutamide and 2Gy radiation on PARP cleavage in AR-insensitive PC3s and AR-sensitive LNCaP prostate models and osteoblastic bone model SJSA-1. Models were treated with 10 μM Abiraterone, Enzalutamide or DMSO 1 or 24 hours before radiation with 2Gy. Samples were then harvested 1,24 and 48 hours post-radiation and expression levels measured *via* Western blot. β -Actin was used as a loading control. (n=3).

As well as changes in the proportion of cells in S and G2, increased levels of PARP cleavage were also observed, with increased PARP cleavage following pre-treatment with Abiraterone or Enzalutamide one or 24 hours before 2 Gy radiation (**Figure 14**). Comparison of Abiraterone and Enzalutamide again showed increased levels of PARP cleavage following abiraterone treatment compared to Enzalutamide. Pre-treatment 24 hours before radiation was shown to be more effective at inducing apoptosis compared to one-hour pre-treatment, with increased PARP cleavage levels observed. Combination treatment of our AR-insensitive prostate model PC3 was again shown to have no impact on PARP-cleavage levels over radiation alone.

## Discussion

Abiraterone acetate and Enzalutamide have seen significant clinical success in an MCRPC setting (4, 5). However, a lack of comparative studies in a prospective, randomized, controlled trial has led to the selection of Abiraterone or Enzalutamide being primarily based on patient factors and side effect profiles.

Reports into the ‘additive’ or ‘synergistic’ nature of Abiraterone and Enzalutamide in combination with radiation in a castration-resistant setting have so far been inconclusive. Several reports have suggested an additive effect (defined as the interaction of Abiraterone or Enzalutamide with radiation being equal to the sum of the two added separately) (19, 20), while others suggest a synergistic effect (defined as the interaction of Abiraterone or Enzalutamide with radiation exceeding the sum of their separate effects) (13, 21–23). We have shown that irrespective of AR status, treatment with Abiraterone or Enzalutamide exerts a significant cytotoxic and additive effect. Which was further supported by observed increases in DNA damage. The reasons behind this effect in AR-insensitive models remains unclear, but may be a consequence of the potential effect of these inhibitors on other signaling mechanisms that can bypass AR signaling such as the glucocorticoid receptor (23). Only in our AR-sensitive LNCaP model was there a synergistic radiosensitive effect with Enzalutamide.

However, Abiraterone was shown to confer a synergistic radiosensitivity effect in all our androgen-insensitive, androgen-sensitive and osteoblastic bone models regardless of AR status, suggesting, as has been previously eluded to (24) the presence of an alternative mechanism of action not dependent on AR inhibition. This potential alternative mechanism of Abiraterone has wider implications, being not only a promising drug for AR-insensitive prostate cancer but Abiraterone may also prove to be beneficial in other malignancies apart from PC.

The interplay between the AR and DNA repair remains a topic of much debate, with previous reports discovering the presence of an AR-mediated transcriptome, leading to the upregulation of various DNA repair genes (12, 13). This in theory suggests that AR-suppression should lead to down-regulation of these genes and thus the enhancement of radiation co-treatment. Our results support this theory, as we have shown in an AR-sensitive setting, that treatment with commonly clinically used AR inhibitors of different modalities i.e., directly (Enzalutamide) or indirectly (Abiraterone) leads to the suppression of key DSB DNA repair pathways such as NHEJ and HR and can be seen to correlate with levels of AR suppression as observed by decreased PSA expression levels. Reductions in HR repair can also be explained in part by shifts in the cell cycle, with Abiraterone and Enzalutamide treatment leading to decreased S and G2 phase, which has also previously been suggested by Zhang et al. (22). This suggests that AR suppression can potentially impact HR repair in both a direct and an indirect manner. Furthermore, suppression of these key repair pathways leads to increased levels of cell death *via* apoptosis as shown by increased levels of PARP-cleavage, supporting the use of these agents clinically as a monotherapy.

Importantly, we have also shown, that downregulation of these key DNA repair genes was conserved when AR suppression through Abiraterone or Enzalutamide is combined with 2Gy radiation (a standard clinical fractionated dose). Thus, supporting the suggestion (25) that it is this key downregulation of key DSB DNA repair pathways that is responsible for our observed radiosensitizing effects with radiation. Our comparisons between one-hour pre-treatment and 24-hour pre-treatment have also shown that for maximal impact, complete AR suppression should be achieved before radiation over concurrent treatment, as even one-hour post-radiation, where DNA damage should be at its maximum, we observed decreased levels of both key NHEJ and HR proteins.

This observed impact on key DSB repair genes raises interest in the potential enhancement of these effects through synthetic lethality approaches, with mounting evidence supporting the combination of inhibiting both the AR and the PARP pathway (26–29). The potential combination of AR suppression and DNA damage response (DDR) inhibitors to increase clinical efficacy is not only limited to PARP inhibition. Several papers have also linked increased cellular toxicity to combinations with ATR inhibitors (30) and Chk1/2 inhibition (31). However, whether this can translate into a clinical setting requires further testing, as, although studies have demonstrated a manageable safety profile (32) there are conflicting reports regarding the clinical efficacy of PARP and AR inhibitor combinations (33, 34).

Regarding the question of selection of Abiraterone or Enzalutamide, our results to date have suggested from a purely biological perspective the increased cytotoxic benefit of Abiraterone over Enzalutamide. This was also shown to be the case mechanistically, with Abiraterone being significantly more impactful on the downregulation of key DNA repair pathway proteins examined (RAD51 and DNA-PK) over Enzalutamide, with this downregulation also occurring at earlier timepoints. Our results have also shown Abiraterone is more effective at inducing cell death than Enzalutamide as observed through increased PARP-cleavage. However, although our results support the preference of the selection of Abiraterone over Enzalutamide, it is important to consider that this is in a strictly *in vitro* setting and does not accurately represent the tumor microenvironment underpinning patients response, which could potentially affect the outcomes. Recent studies have also suggested a sequencing approach of Abiraterone followed by Enzalutamide may result in an increased clinical benefit (35), although many centres adopt an either/or approach with regards to the selection of these two agents. There has been an increase in the use of both agents in the frontline setting which in turn will lead to increased number of patients receiving high dose radiotherapy to the prostate in combination with these agents. This has been amplified to negate any immunosuppressive impact of the previous standard of care, docetaxel.

In conclusion, we have demonstrated that while Abiraterone and Enzalutamide have an additive cytotoxic effect regardless of AR status, radiosensitization in an AR-sensitive setting is due to downregulation of multiple key DNA repair pathways such as NHEJ and HR, which may also be mediated by cell cycle distribution changes. Furthermore, comparisons of Abiraterone versus Enzalutamide have shown Abiraterone to be significantly more effective in terms of inhibiting key DNA repair proteins and cell death than Enzalutamide, providing a rationale of its selection over Enzalutamide in a clinical setting should side effect profiles not be a consideration.

## Data Availability Statement

The raw data supporting the conclusions of this article will be made available by the authors, without undue reservation.

## Author Contributions

Conceptualization, AC and KP. Methodology, TW, VD, KR, AC, and KP. Investigation, TW, AA, and VD. Data curation, TW and VD. Writing—original draft preparation, TW, AC, and KP. Writing—review and editing, TW, VD, AC, and KP. Supervision, VD, AC, and KP. Funding acquisition, AC and KP. All authors contributed to the article and approved the submitted version.

## Funding

This research was funded by the Belfast-Manchester Movember Centre of Excellence (CE013_2-004), funded in partnership with Prostate Cancer UK. TW was supported by a studentship from the Northern Ireland Department of the Economy. VD is supported by the LFT Charitable Trust.

## Conflict of Interest

The authors declare that the research was conducted in the absence of any commercial or financial relationships that could be construed as a potential conflict of interest.
