# Supplementary material for: Abiraterone In Vitro Is Superior to Enzalutamide in Response to Ionizing Radiation
Source: Front Oncol. 2021 Jul 21;11:700543. doi: 10.3389/fonc.2021.700543 (PMC8335570; doi:10.3389/fonc.2021.700543)
Supplement: Supplementary file 1 [file Table_1.docx]

Supplementary Material

# Supplementary Figures and Tables

**Supplementary Table 1-** Fold sensitivity increase relative to DMSO of MTT values across cell lines ± SEM

| **Fold increase** | **Treatment** | **LNCaP** | **SJSA-1** | **PC3** |
| --- | --- | --- | --- | --- |
|  | DMSO | 1.0 | 1.0 | 1.0 |
|  | Enz | 7.6 | 3.1 | 3.5 |
|  | Abi | 15.8 | 6.6 | 6.6 |

**Supplementary Table 2**- Cell cycle distribution % for **Figure 9-** AR-sensitive LNCaP prostate model, AR- insensitive PC3 prostate model and osteoblastic bone model SJSA-1. Cells were treated with 10uM Abiraterone, Enzalutamide or DMSO, fixed 1h, 24h and 48h post-treatment and stained with PI/RNaseA for 30 minutes before the cell-cycle profile was determined by flow cytometry. Error bars are standard error of the mean (SEM) (n=3).

| **LNCaP** | **Sub-G1** | **±** | **G1** | **±** | **S** | **±** | **G2** | **±** |
| --- | --- | --- | --- | --- | --- | --- | --- | --- |
| **Con** | 7.17% | 1.13% | 59.89% | 2.26% | 13.65% | 0.95% | 22.52% | 0.95% |
| **DMSO 1h** | 4.98% | 0.47% | 61.07% | 2.13% | 13.33% | 1.10% | 23.41% | 2.10% |
| **Enz 1h** | 7.24% | 0.89% | 60.41% | 2.26% | 14.25% | 1.06% | 21.43% | 1.06% |
| **Abi 1h** | 5.38% | 1.46% | 61.20% | 0.43% | 13.44% | 0.47% | 23.13% | 0.47% |
| **DMSO 24h** | 8.83% | 0.74% | 59.14% | 1.66% | 15.41% | 1.61% | 20.35% | 1.61% |
| **Enz 24h** | 6.20% | 0.65% | 58.71% | 2.32% | 14.03% | 1.16% | 24.33% | 2.16% |
| **Abi 24h** | 16.22% | 0.68% | 54.57% | 0.52% | 11.44% | 0.60% | 20.84% | 0.60% |
| **DMSO 48h** | 9.87% | 1.44% | 55.09% | 1.06% | 14.89% | 0.13% | 23.47% | 0.13% |
| **Enz 48h** | 15.84% | 1.61% | 57.14% | 1.50% | 14.79% | 1.65% | 16.37% | 1.65% |
| **Abi 48h** | 31.60% | 1.85% | 46.56% | 0.74% | 13.92% | 1.68% | 12.29% | 1.68% |
|  |  |  |  |  |  |  |  |  |
| **PC3** | **Sub-G1** | **±** | **G1** | **±** | **S** | **±** | **G2** | **±** |
| **Con** | 5.11% | 3.13% | 59.64% | 2.26% | 17.72% | 2.26% | 18.62% | 0.95% |
| **DMSO 1h** | 7.36% | 0.47% | 61.35% | 2.13% | 17.71% | 2.13% | 15.01% | 2.10% |
| **Enz 1h** | 7.38% | 1.89% | 62.17% | 2.26% | 17.01% | 1.26% | 14.94% | 1.06% |
| **Abi 1h** | 6.86% | 1.46% | 60.34% | 0.43% | 15.55% | 0.43% | 18.44% | 0.47% |
| **DMSO 24h** | 7.36% | 2.74% | 61.35% | 2.66% | 17.71% | 1.66% | 15.01% | 1.61% |
| **Enz 24h** | 7.38% | 1.65% | 62.17% | 2.32% | 17.01% | 2.32% | 14.94% | 2.16% |
| **Abi 24h** | 6.86% | 0.68% | 60.34% | 0.52% | 15.55% | 0.12% | 18.44% | 0.60% |
| **DMSO 48h** | 7.36% | 1.44% | 61.35% | 2.06% | 17.71% | 2.06% | 15.01% | 0.13% |
| **Enz 48h** | 7.38% | 1.61% | 62.17% | 1.50% | 17.01% | 1.50% | 14.94% | 1.65% |
| **Abi 48h** | 6.86% | 2.85% | 60.34% | 0.74% | 15.55% | 0.74% | 18.44% | 1.68% |
|  |  |  |  |  |  |  |  |  |
| **SJSA-1** | **Sub-G1** | **±** | **G1** | **±** | **S** | **±** | **G2** | **±** |
| **Con** | 2.67% | 3.13% | 85.56% | 2.26% | 6.38% | 0.26% | 5.62% | 0.95% |
| **DMSO 1h** | 3.02% | 0.47% | 83.33% | 2.13% | 7.72% | 0.13% | 6.26% | 0.10% |
| **Enz 1h** | 4.57% | 1.89% | 82.25% | 2.26% | 8.43% | 0.26% | 5.11% | 0.06% |
| **Abi 1h** | 4.57% | 1.46% | 82.25% | 0.43% | 8.43% | 0.43% | 5.11% | 0.47% |
| **DMSO 24h** | 3.02% | 2.74% | 83.33% | 2.66% | 7.72% | 0.66% | 6.26% | 0.61% |
| **Enz 24h** | 4.57% | 1.65% | 82.25% | 2.32% | 8.43% | 0.32% | 5.11% | 0.16% |
| **Abi 24h** | 4.57% | 0.68% | 82.25% | 0.52% | 8.43% | 0.52% | 5.11% | 0.60% |
| **DMSO 48h** | 3.51% | 1.44% | 84.00% | 2.06% | 8.25% | 0.06% | 4.43% | 0.13% |
| **Enz 48h** | 3.73% | 1.61% | 83.47% | 1.50% | 7.99% | 0.50% | 5.08% | 0.65% |
| **Abi 48h** | 3.73% | 2.85% | 83.47% | 0.74% | 7.99% | 0.74% | 5.08% | 0.68% |

**Supplementary Table 3**- Cell cycle distribution % ± SEM for **Figure** 11- AR-sensitive LNCaP prostate model, AR- insensitive PC3 prostate model and osteoblastic bone model SJSA-1. Cells were treated with 10uM Abiraterone, Enzalutamide or DMSO 1 or 24 hours before radiation with 2gy. Post radiation cells were fixed 1h, 24h and stained with PI/RNaseA for 30 minutes before the cell-cycle profile was determined by flow cytometry. Error bars are standard error of the mean (SEM) (n=3).

| **LNCaP 1h Pre** | **Sub-G1** | **±** | **G1** | **±** | **S** | **±** | **G2** | **±** |
| --- | --- | --- | --- | --- | --- | --- | --- | --- |
| **Con** | 2.35% | 0.34% | 53.91% | 0.22% | 16.43% | 0.0186 | 27.89% | 0.0183 |
| **DMSO 1h** | 1.67% | 0.55% | 54.42% | 0.78% | 16.36% | 1.11% | 28.15% | 0.0142 |
| **Enz 1h** | 1.76% | 0.44% | 54.50% | 1.52% | 15.91% | 1.13% | 28.57% | 0.023 |
| **Abi 1h** | 1.40% | 0.25% | 52.24% | 3.20% | 17.26% | 0.48% | 29.74% | 0.0352 |
| **DMSO 24h** | 2.48% | 0.66% | 68.09% | 0.16% | 7.18% | 1.71% | 22.49% | 0.0092 |
| **Enz 24h** | 2.23% | 0.48% | 69.87% | 0.98% | 3.68% | 0.45% | 24.36% | 0.0102 |
| **Abi 24h** | 3.52% | 0.46% | 63.50% | 2.52% | 5.46% | 1.03% | 27.86% | 0.0204 |
| **DMSO 48h** | 3.44% | 0.70% | 53.68% | 0.22% | 13.85% | 1.26% | 30.04% | 0.0139 |
| **Enz 48h** | 3.16% | 0.77% | 66.31% | 0.46% | 7.29% | 0.49% | 23.79% | 0.0028 |
| **Abi 48h** | 8.39% | 1.27% | 61.03% | 1.40% | 9.06% | 1.26% | 22.68% | 0.0101 |

| **LNCaP 24h Pre** | **Sub-G1** | **±** | **G1** | **±** | **S** | **±** | **G2** | **±** |
| --- | --- | --- | --- | --- | --- | --- | --- | --- |
| **Con** | 2.56% | 0.02% | 47.86% | 0.06% | 14.89% | 0.0064 | 36.92% | 0.0129 |
| **DMSO 1h** | 2.19% | 0.49% | 55.49% | 2.86% | 12.51% | 0.82% | 31.71% | 0.0226 |
| **Enz 1h** | 1.47% | 0.30% | 63.32% | 0.04% | 7.91% | 0.56% | 28.60% | 0.0038 |
| **Abi 1h** | 2.57% | 0.26% | 64.63% | 1.03% | 6.20% | 0.39% | 27.49% | 0.0069 |
| **DMSO 24h** | 1.29% | 0.35% | 69.50% | 0.60% | 4.62% | 1.14% | 25.37% | 0.0024 |
| **Enz 24h** | 0.97% | 0.19% | 74.28% | 0.38% | 1.19% | 0.27% | 23.79% | 0.0007 |
| **Abi 24h** | 2.40% | 0.45% | 72.42% | 1.35% | 1.93% | 0.13% | 23.60% | 0.007 |
| **DMSO 48h** | 3.10% | 0.57% | 61.34% | 0.60% | 9.97% | 0.12% | 27.30% | 0.0068 |
| **Enz 48h** | 1.45% | 0.27% | 73.44% | 1.43% | 2.82% | 0.34% | 22.76% | 0.0067 |
| **Abi 48h** | 5.25% | 1.75% | 72.25% | 0.06% | 2.92% | 0.27% | 20.26% | 0.0203 |

| **PC3 24h Pre** | **Sub-G1** | **±** | **G1** | **±** | **S** | **±** | **G2** | **±** |
| --- | --- | --- | --- | --- | --- | --- | --- | --- |
| **Con** | 1.43% | 0.18% | 55.09% | 0.75% | 22.32% | 0.00435 | 24.75% | 0.0175 |
| **DMSO 1h** | 1.17% | 0.59% | 54.58% | 1.10% | 21.58% | 0.53% | 25.56% | 0.0089 |
| **Enz 1h** | 1.13% | 2.22% | 54.40% | 1.57% | 23.00% | 1.96% | 24.30% | 0.0273 |
| **Abi 1h** | 1.73% | 1.01% | 52.91% | 0.92% | 23.12% | 0.17% | 25.31% | 0.0081 |
| **DMSO 24h** | 1.51% | 1.12% | 43.77% | 0.37% | 27.36% | 2.63% | 31.02% | 0.0472 |
| **Enz 24h** | 1.29% | 1.82% | 43.85% | 0.96% | 26.19% | 3.47% | 32.16% | 0.066 |
| **Abi 24h** | 1.88% | 1.27% | 46.62% | 1.54% | 25.69% | 1.95% | 29.66% | 0.1354 |
| **DMSO 48h** | 18.47% | 3.62% | 58.48% | 3.81% | 19.28% | 4.99% | 22.53% | 0.057 |
| **Enz 48h** | 3.41% | 2.08% | 58.38% | 2.24% | 20.60% | 3.39% | 20.36% | 0.0337 |
| **Abi 48h** | 3.18% | 0.31% | 57.97% | 0.18% | 19.44% | 1.26% | 22.13% | 0.0226 |

| **SJSA-1 24h Pre** | **Sub-G1** | **±** | **G1** | **±** | **S** | **±** | **G2** | **±** |
| --- | --- | --- | --- | --- | --- | --- | --- | --- |
| **Con** | 1.03% | 0.27% | 60.77% | 0.05% | 18.05% | 0.0099 | 20.48% | 0.44% |
| **DMSO 1h** | 0.82% | 0.25% | 52.97% | 1.49% | 23.26% | 1.52% | 23.38% | 0.62% |
| **Enz 1h** | 0.76% | 0.18% | 53.07% | 1.92% | 23.90% | 0.67% | 22.70% | 1.85% |
| **Abi 1h** | 1.14% | 0.12% | 51.56% | 3.99% | 23.36% | 2.17% | 24.24% | 2.22% |
| **DMSO 24h** | 0.86% | 0.20% | 67.19% | 0.20% | 15.37% | 0.81% | 17.01% | 1.24% |
| **Enz 24h** | 0.94% | 0.52% | 67.11% | 0.64% | 15.02% | 0.63% | 17.33% | 0.13% |
| **Abi 24h** | 1.40% | 0.39% | 66.80% | 1.48% | 16.12% | 0.37% | 16.12% | 1.89% |
| **DMSO 48h** | 3.18% | 0.52% | 74.76% | 1.94% | 10.09% | 1.23% | 11.98% | 3.69% |
| **Enz 48h** | 3.26% | 0.53% | 74.40% | 1.52% | 9.82% | 1.27% | 12.54% | 3.30% |
| **Abi 48h** | 2.35% | 0.78% | 75.38% | 0.69% | 9.33% | 0.71% | 13.53% | 1.20% |
